# Serum exosomal microRNAs combined with alpha‐fetoprotein as diagnostic markers of hepatocellular carcinoma

**DOI:** 10.1002/cam4.1390

**Published:** 2018-03-23

**Authors:** Yurong Wang, Chunyan Zhang, Pengjun Zhang, Guanghong Guo, Tao Jiang, Xiumei Zhao, Jingjing Jiang, Xueliang Huang, Hongli Tong, Yaping Tian

**Affiliations:** ^1^ Core Laboratory of Translational Medicine State Key Laboratory of Kidney Disease Chinese PLA General Hospital 28 Fu‐Xing Road Beijing 100853 China; ^2^ School of Medicine Nankai University 94 Weijin Road Tianjin 300071 China; ^3^ Department of Clinical Biochemistry Chinese PLA General Hospital 28 Fu‐Xing Road Beijing 100853 China

**Keywords:** Alpha‐fetoprotein, biomarker, exosomes, hepatocellular carcinoma, microRNA

## Abstract

Exosomal microRNAs have recently been studied as the potential diagnostic marker for various malignancies, including hepatocellular carcinoma (HCC). The aim of this study was to investigate serum exosomal microRNA profiles as HCC diagnostic marker. Transmission electron microscopy and Western blot were used to identify serum exosomes. Deep sequencing was performed to screen differentially expressed microRNAs between HCC (*n* = 5) and liver cirrhosis (LC,* n* = 5) groups. Three upregulated and two downregulated microRNAs were selected for qPCR analysis. The levels of selected microRNAs were normalized to Caenorhabditis elegans miR‐39 microRNA mimics. Serum exosomal level of miR‐122, miR‐148a, and miR‐1246 was further analyzed and significantly higher in HCC than LC and normal control (NC) groups (*P* < 0.001), but not different from chronic hepatitis group (*P* > 0.05). The receiver operating characteristic curve was used to evaluate the diagnostic performance of candidate microRNAs. Area under the curve (AUC) of miR‐148a was 0.891 [95% confidence interval (CI), 0.809–0.947] in discriminating HCC from LC, remarkably higher than alpha‐fetoprotein (AFP) (AUC: 0.712, 95% CI: 0.607–0.803). Binary logistic regression was adopted to establish the diagnostic model for discriminating HCC from LC. And the combination of miR‐122, miR‐148a, and AFP increased the AUC to 0.931 (95% CI, 0.857–0.973), which can also be applied for distinguishing early HCC from LC. miR‐122 was the best for differentiating HCC from NC (AUC: 0.990, 95% CI, 0.945–1.000). These data suggest that serum exosomal microRNAs signature or their combination with traditional biomarker may be used as a suitable peripheral screening tool for HCC.

## Introduction

Hepatocellular carcinoma (HCC) remains one of the most common and deadliest cancers worldwide 1, 2. The application of serum alpha‐fetoprotein (AFP) in HCC screening is controversial, with a low sensitivity range from 39% to 65% 3. Although the improvement of imaging techniques has made small nodular lesions less than 1 cm detectable, repeating MRI and CT scans represents a problem of an economic burden. Furthermore, small nodules in the cirrhotic liver may still be a challenge for the above noninvasive detection methods. Considering that liver cirrhosis (LC) increases the risk of HCC at a rate of approximately 3% annually, 4 seeking reliable biomarkers would be valuable for screening and diagnosing HCC with LC, investigation on which is still being highly valued according to the guidelines of EASL‐EORTC and AASLD 5, 6, 7.

MicroRNAs are small, noncoding RNA that regulate protein expression through target mRNAs degradation or translational inhibition, involved in a variety of processes, such as cell proliferation, cell apoptosis, genomic instability, tumor metastasis, and immune response 8, 9, 10. Dysregulation of microRNAs has been reported in various human malignancies, including HCC. Because of the convenience and reproducible of sample collecting, cell‐free circulating microRNA is being actively researched as noninvasive markers for cancer. And it is worth mentioning more than 100 clinical trials are ongoing for incorporating microRNAs as biomarkers in lung cancer, breast cancer, leukemias, lymphomas, neuroblastoma, ovarian cancer, and prostate cancer 11. In addition to usage in cancer diagnosis, microRNAs can also be applied to the histological type, disease prognosis, and clinical effect. In particular, several circulating microRNAs have been reported as candidates for HCC diagnosis and prognosis 12.

Exosomes are small membranous vesicles with 30–150 nm of diameter, which derive from internal multivesicular bodies and exist in many kinds of body fluids such as serum, urine, and breast milk, carrying cell‐specific protein, mRNA, and microRNA 13. Exosomes have recently been reported to actively participate in cell–cell communication, tumorigenesis and development, and targeted delivery of drugs 14, 15, 16. Because of the structure of “lipid raft domains,” exosomes can protect its containing microRNA from degradation of RNase to stably exist in circulation 17. MicroRNAs in exosomes have been shown to be similar to those in their cells of origin, suggesting their value as a marker for disease diagnosis 18. Report from Wang et al. 19 revealed that serum exosomal level of miR‐21 was higher than that in the serum of HCC, chronic hepatitis, and healthy individuals, suggesting exosomes as the main source of serum microRNA. Sohn, Won, and coworkers detected serum exosomal microRNA in patients with chronic hepatitis B (CHB), LC and HCC with CHB, and discovered that a panel of eight exosomal microRNAs, including miR‐122, were significantly different between HCC and CHB or LC groups 20. Given the above, exosomal microRNA as a biomarker for HCC diagnosis is feasible, and so far, few systematic research on this topic has been carried out.

In this study, we detected the expression level of exosomal microRNAs in LC and HCC with LC patients through microRNA deep sequencing. Cirrhotic patients, instead of healthy individuals, were chosen as controls because they represent the population at high risk for HCC. And RT‐qPCR was adopted to verify the candidate microRNA in independent validation cohorts which consist of normal control (NC), chronic hepatitis (CH), LC, and HCC individuals. We aimed to establish an optimal biomarker for HCC diagnosis and screening using serum exosomal microRNA or their combination with the traditional marker.

## Material and Methods

### Clinical samples and data collection

ALL serum samples were obtained between December 2015 and August 2016 from individuals with and without liver pathologies (NC, CH, LC, and HCC), who were admitted to or hospitalized in Hepatobiliary Surgery Department or Gastroenterology Department in the Chinese PLA General Hospital. The study was approved by Chinese PLA General Hospital's Ethics Committee. All subjects involved in this study provided written informed consents. This study was designed as a two‐stage investigation with a discovery step performed by deep sequencing, obtaining a panel of aberrantly expressed exosomal microRNA between LC (*n* = 5) and HCC with LC (*n* = 5). The validation stage was conducted on two independent cohorts of patients. The total number listed as follows is 235: 64 cases with NC, 50 cases with CH, 53 cases with LC, and 68 cases with HCC. Exosomes and exosomal RNA from all of the above cases were isolated and extracted. Diagnosis of HCC was based on histopathology, and none of those had undergone cancer‐directed treatments (such as surgery, chemotherapy, radiotherapy, and so on) or had another kind of cancer. Liver cirrhosis and chronic hepatitis were diagnosed according to clinical characteristics, laboratory testing, and imaging results of viral infection and hepatic damage. All of LC patients were confirmed by imaging techniques without a vascular enhancing mass. Normal subjects were the individuals in normal physical condition without liver disease or viral infection. Tumor staging was according to AJCC/UICC (2010) TNM staging system for HCC. Early HCC has been defined as single lesion <5 cm in diameter or <3 lesions, each <3 cm in diameter). Child‐Pugh Class was adopted for evaluation of liver function: A (5–6 scores), B (7–9 scores), C (10–15 scores). The values of alanine aminotransferase (ALT), aspartate aminotransferase (AST), alkaline phosphatase (ALP), glutamyl aminotransferase (GGT), albumin, total bilirubin (TB), and AFP were detected and recorded.

### Serum exosomes isolation

Referring to the previous studies, we established a protocol for isolating serum exosomes and described in details as below. Peripheral blood from all subjects was collected in tubes with separating gel and clot activator, and then centrifuged at 3000 *g* for 10 min at room temperature. The supernatants were obtained and transferred to another clean tube and stored at −80°C until RNA extraction. Polyethylene glycol (PEG) 6000 (Sigma‐Aldrich, St Louis, MO) of 8% concentration was used to isolate exosomes from the serum 21. First, serum was thawed and centrifuged at 3000 *g* for 15 min to remove the cell debris and then filtrated through 0.22‐*μ*m filter to remove larger vesicles. And 900 *μ*L serum was mixed with 300 *μ*L PEG 6000 of 32% concentration and then kept at 4°C overnight. The mixture was centrifuged at 12,000 *g* for 30 min. The protein‐rich supernatants were discarded, and the exosome‐rich pellets were resolved by 200 *μ*L 0.01 mol/L phosphate‐buffered saline (PBS) and stored at −80°C until use.

### Transmission electron microscopy

The exosome‐rich pellets were dissolved in 0.01 mol/L PBS, and 10 *μ*L of the suspension was dropped onto a carbon‐coated copper grid and allowed stand for 5 min. The grid was then drained using the filter paper. A drop of 2% uranyl acetate was dropped onto the grid for 1 min and then drained by the filter paper. Dry the grid at room temperature for several minutes and then observe using a transmission electron microscope (TEM) at 80 K electron volts.

### Western blot

Exosomal protein extracted from serum was electrophoresed and electroblotted following a standard blotting protocol. Specific primary antibodies were used, and their dilutions were as follows: mouse anti‐CD63 (Abcam, Cambridge, MA, 1:500), rabbit antiflotillin 1 (Abcam, 1:1000), rabbit anti‐CD9 (Abcam, 1:2000). HRP‐conjugated goat anti‐mouse IgG and goat anti‐rabbit IgG (Proteintech Group, Chicago, USA, both diluted 1:3000) were used as the secondary antibodies.

### Exosomal RNA isolation and microRNA sequencing

The total exosomal RNA was extracted from serum using miRNeasy Mini Kit (Qiagen, Hilden, Germany) according to the manufacturer's protocols. In brief, Qiazol lysis buffer was added to exosomes, and the homogenate was placed at room temperature for 5 min. Then, 5 *μ*L 0.26 nmol/L cel‐miR‐39 mimic was added to each sample followed by adding chloroform and vortexing for 15 sec. Subsequent steps were conducted as described in the manufacturer's protocol. Serum exosomal microRNA profiling was performed using Illumina HiSeq 2000 sequencing technology by Kangchen Bio‐tech Inc (Shanghai, China). These sequence data have been submitted to the DDBJ/EMBL/GenBank databases under accession number GSE104251.

### TaqMan microRNA assay for individual exosomal microRNAs

MicroRNA expression in serum exosomes from all subjects was assessed by RT‐qPCR. For cDNA synthesis, TaqMan microRNA Reverse Transcription Kit and TaqMan microRNA‐specific primers (Applied Biosystems, CA, USA) were used following the manufacturer's protocol. Reverse transcription was conducted in a scaled down reaction volume of 7.5 *μ*L, including 2.08 *μ*L of RNase‐free water, 0.075 *μ*L of dNTPs with dTTP, 0.75 *μ*L 10× RT buffer, 0.5 *μ*L of multiscribe reverse transcriptase, 0.095 *μ*L of RNase inhibitor, 1.5 *μ*L of microRNA‐specific stem‐loop RT primer, and 2.5 *μ*L (25 ng) of total RNA template. Reverse transcription reaction was then performed under the following conditions: 16°C for 30 min, 42°C for 30 min, and 85°C for 5 min.

Thereafter, TaqMan microRNA assay (Applied Biosystem) was adopted to quantify individual exosomal microRNA as described previously 22. Briefly, 20 *μ*L reaction system was prepared as follows: 10 *μ*L TaqMan Universal PCR Master Mix, 1 *μ*L TaqMan microRNA assay mix, and 1.33 *μ*L of RT products, and 7.67 *μ*L nucleotide‐free water. qPCR was performed on ABI PRISM 7300 system at the following condition: 50°C for 2 min, 95°C for 5 min, and 40 cycles of 95°C for 15 sec and 60°C for 1 min. All qPCR reactions were conducted in duplicate, and the Ct values greater than 35 were defined as 35. The expression levels of microRNAs were calculated by the 2^−ΔCt^ method as described previously 22.

### Statistical analysis

The significance of serum exosomal microRNAs between different groups was determined by Mann–Whitney *U* test or Kruskal–Wallis *H* test. MedCalc 12.7.0 software was used to obtain receiver operating characteristic curves (ROC) and calculate the area under the curve (AUC). The diagnostic models for differentiating HCC were established through binary logistic regression with candidate exosomal microRNAs and main HCC‐related biochemical indicators such as ALT, AST, ALP, GGT, albumin, TB, and AFP as variables. And AUC comparisons between the different indexes, including single index and composite index, were conducted through the area *z*‐score test. The correlations between the levels of exosomal microRNA and laboratory parameters were performed by linear regression analysis. *P* values less than 0.05 were considered as statistically significant. And all statistics were calculated by means of SPSS 19.0 software.

## Results

### Identification of serum exosomes

**Figure 1:**
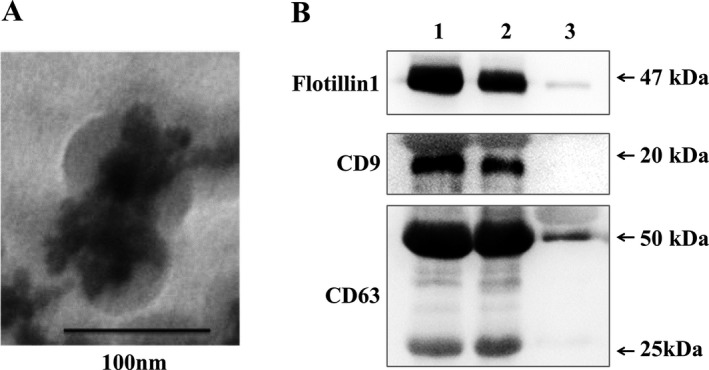
Identification of exosomes from serum. (A) Morphological identification of serum exosomes by transmission electron microscopy. (B) Biomarker detection of serum exosomes by Western blotting. Lane 1, 2, and 3: the serum exosome fractions from HCC patient 1, 2, and the exosome‐free serum of HCC patient 1.

The morphological feature of serum exosomes was observed by transmission electron microscope and is shown in Figure 1A. And protein markers of exosomes (CD9, CD63, and flotillin 1) are detected by Western blot in Figure 1B. The results suggested exosomes can be successfully extracted from serum through this protocol for the subsequent experiment.

### Serum exosomal microRNA profiling and data analysis

To investigate serum exosomal microRNA accurately, microRNA sequencing was performed in each sample from LC (*n* = 5) and HCC (*n* = 5) groups using Illumina HiSeq 2000 technology. Basic information of these samples is shown in Table S1. The data indicated that a total of 1244 microRNAs could be detectable. By comparing the exosomal microRNA profiles between the two groups, there were 11 microRNAs significantly upregulated (10 known and one novel microRNA), 86 significantly downregulated, and 1147 undifferentiated expressed (Fig. S1). As shown in Table 1, the top 10 upregulated and downregulated microRNAs were listed with fold change >1.5 and *P* < 0.05. Additionally, based on the condition of tags per million reads >400, a panel of exosomal microRNAs with relative higher abundance was selected for the subsequent microRNA RT‐qPCR test: miR‐122, miR‐148a, miR‐1246, miR‐486, and miR‐584.

**Table 1:** Upregulated and downregulated serum exosomal microRNAs of HCC compared to LC. Fold change was calculated as mean expression of microRNA in HCC group compared versus that in LC group. The top ten microRNAs upregulated or downregulated with fold change >1.5 and *P* < 0.05 are listed, respectively. The five microRNAs (with tags per million reads >400) highlighted with italics represent the candidates selected for subsequent validation

Upregulation		Downregulation	
microRNA	Fold change	microRNA	Fold change
*miR‐122‐5p*	2.9	miR‐215‐5p	8.4
miR‐455‐5p	2.7	miR‐4443	2.6
miR‐192‐5p	2.4	*miR‐486‐5p*	2.6
miR‐100‐5p	2.2	miR‐423‐5p	2.4
*miR‐1246‐5p*	2.2	let‐7d‐3p	2.3
*miR‐148a‐3p*	1.7	miR‐203a‐3p	2.1
miR‐323b‐3p	1.7	miR‐16‐2‐3p	1.9
miR‐148a‐5p	1.7	miR‐342‐5p	1.8
miR‐194‐3p	1.6	miR‐101‐3p	1.8
miR‐452‐5p	1.6	*miR‐584‐5p*	1.8

### Candidate exosomal microRNA selection and preliminary evaluation

**Figure 2:**
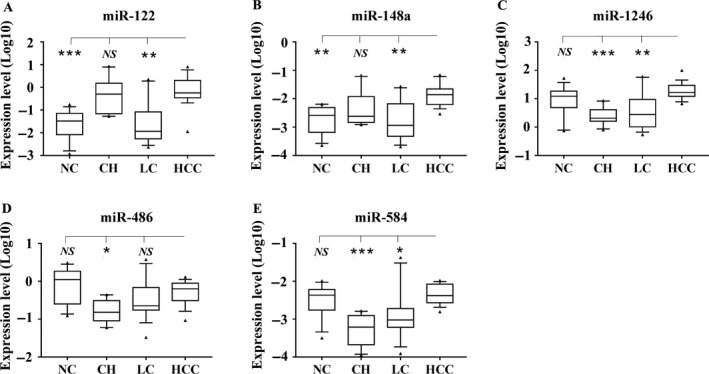
The expression of serum exosomal microRNAs in NC, CH, LC, and HCC groups. Expression level of miR‐122 (A), miR‐148a (B), miR‐1246 (C), miR‐486 (D), and miR‐584 (E) in an independent set of serum exosomes from NC (*n* = 14), CH (*n* = 10), LC (*n* = 13), and HCC (*n* = 18) was quantified using real‐time qPCR. Each qPCR was carried out in duplicate. Expression levels of selected microRNAs were normalized to cel‐miR‐39 and presented as log10 (2^−ΔCt^). Data are shown as the median values (10–90 percentile ranges). A Mann–Whitney or Kruskal–Wallis test was used to determine statistical significance. ***P < 0.001, **P < 0.01, *P < 0.05, NS: nonsignificant.

To screen the candidate serum exosomal microRNAs (miR‐122, miR‐148a, miR‐1246, miR‐486, and miR‐584), preliminary experiments were conducted on a second set of exosome samples, including subjects with NC (*n* = 14), CH (*n* = 10), LC (*n* = 13), and HCC (*n* = 18). As shown in Figure S2, we can observe that the Ct values of the same miRNA between samples are very close, so repeatability of the detection is good, can be used in subsequent experiments. The clinical baseline characteristics of these subjects are listed in Table S2. The results showed that miR‐122 (Fig. 2A) and miR‐148a (Fig. 2B) levels were significantly higher in HCC than in LC and NC groups (*P* < 0.01), but not apparently different from CH group. The expression level of miR‐1246 (Fig. 2C) was significantly higher in HCC than in LC (*P* < 0.01) and CH groups (*P* < 0.001), while not different from NC group. miR‐486 expression level was not significantly different between LC and HCC groups (Fig. 2D). And miR‐584 level was apparently higher in HCC than LC group (Fig. 2E). miR‐486 and miR‐584 were excluded due to their inconsistent results between qPCR analysis and sequencing analysis.

### Identification of exosomal microRNAs as potential diagnostic biomarker for HCC

**Figure 3:**
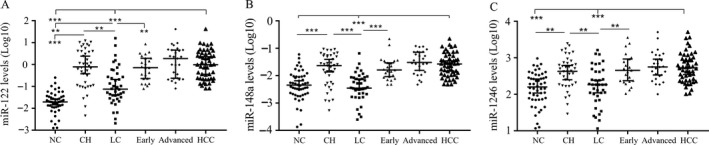
Validation of three serum exosomal microRNAs in an independent samples. Scatter plots of serum exosomal levels (2^−ΔCt^) of miR‐122 (A), miR‐148a (B), and miR‐1246 (C) in NC (*n* = 50), CHB (*n* = 40), LC (*n* = 40), early HCC (*n* = 23), advanced HCC (*n* = 27), and total HCC (*n* = 50). Expression levels of microRNAs were normalized to cel‐miR‐39 and presented as log10 scale. Statistical significance was analyzed by Mann–Whitney or Kruskal–Wallis test. ***P < 0.001, **P < 0.01, *P < 0.05.

We further analyzed miR‐122, miR‐148a, and miR‐1246 in another independent cohort of serum samples, including NC (*n* = 50), CH (*n* = 40), LC (*n* = 40), and HCC (*n* = 50) (with baseline information listed in Table 2). As shown in Figure 3A, miR‐122 expression level in early HCC and advanced HCC group was both significantly higher than in LC and NC groups (*P* < 0.001), but not different from CH group (*P* > 0.05). The expression pattern of miR‐148a (Fig. 3B) and miR‐1246 (Fig. 3C) was similar to miR‐122 in each group.

**Table 2:** Clinical characteristics of 180 cases for validation. Ages and albumin are given as means (SD), and AFP, ALT, ALP, AST, GGT, and TB are given as medians (interquartile range)

Variables	HCC (*n* = 50)	LC (*n* = 40)	CH (*n* = 40)	NC (*n* = 50)	*P*‐value
Age (years)	56.32 ± 9.71	55.13 ± 11.94	51.25 ± 8.46	53.92 ± 8.17	NS
Sex, male/female (*n*)	40/10	25/15	31/9	37/13	NS
Etiology, HBV/HCV/Normal (*n*)	42/2/6	35/2/3	32/8/0	0/0/50	<0.001***
ALT (U/L)	28.50 (16.43–47.33)	21.20 (15.48–32.73)	22.40 (16.53–37.83)	15.20 (11.20–18.80)	<0.001***
AST (U/L)	27.30 (18.43–35.58)	23.50 (18.00–34.60)	20.35 (16.08–26.30)	18.15 (15.38–20.75)	<0.001***
Albumin (g/L)	39.47 ± 3.47	35.50 ± 4.43	44.15 ± 3.09	45.26 ± 2.00	<0.001***
ALP (U/L)	74.80 (61.90–95.78)	81.40 (57.05–99.50)	69.65 (54.85–85.50)	64.75 (52.80–80.38)	0.022*
GGT (U/L)	49.45 (30.13–92.18)	33.25 (17.15–57.20)	31.75 (13.90–51.73)	15.15 (12.53–20.50)	<0.001***
TB (μmol/L)	14.25 (11.43–16.48)	17.45 (12.38–27.65)	10.25 (7.95–12.93)	10.85 (9.05–13.28)	<0.001***
AFP (μg/L)	16.46 (3.39–100.20)	3.31 (2.24–19.00)	2.55 (1.86–3.21)	3.23 (2.29–4.08)	<0.001***
<20/>20 (*n*)	23/27	38/2	40/0	49/1	
Child‐Pugh Class, A/B/C (*n*)	45/5/0	20/18/2	40/0/0	40/0/0	
TNM staging, 1/2/3/4 (*n*)	28/9/13/0	NA	NA	NA	
Histological grade, good/moderate/poor (*n*)	5/38/7	NA	NA	NA	
Portal vein tumor thrombus (*n*)	5	NA	NA	NA	

AFP, alpha‐fetoprotein; ALP, alkaline phosphatase; ALT, alanine aminotransferase; AST, aspartate aminotransferase; CH, chronic hepatitis; GGT, glutamyl aminotransferase; HBV, hepatitis B virus; HCC, hepatocellular carcinoma; HCV, hepatitis C virus; LC, liver cirrhosis; NC: normal, NA, not available; NS, no difference among groups; TB, total bilirubin. *P < 0.05, ***P < 0.001.

**Figure 4:**
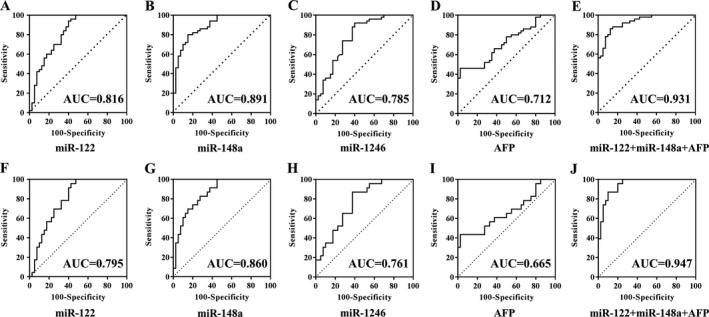
ROC curve analysis of serum exosomal microRNAs to differentiate patients with (early) HCC from LC. A–E and F–J showed the ROC curves of miR‐122, miR‐148a, miR‐1246, AFP, and the combination of miR‐122, miR‐148a, and AFP for HCC (*n* = 50) versus LC (*n* = 40) and early HCC (*n* = 27) versus LC (*n* = 40).

Then, we analyzed the diagnostic performance of the above three serum exosomal microRNAs in discriminating HCC from LC. ROC analysis results showed that the AUC and 95% confidence interval (CI) of miR‐122, miR‐148a, and miR‐1246 was 0.816 [95% CI: 0.720–0.889, *P* < 0.0001), 0.891 (95% CI: 0.809–0.947, *P* < 0.0001), and 0.785 (95% CI: 0.686–0.865, *P* < 0.0001), respectively, in discriminating HCC from LC, and only miR‐148a was significantly higher than AFP 0.712 (95% CI: 0.607–0.803, *P* < 0.0001) (Fig. 4A–D). The exosomal microRNAs and main HCC‐related biochemical indicators (ALT, AST, ALP, GGT, albumin, TB, and AFP) were analyzed through binary logistic regression method. Then, we obtained that the combination of miR‐122, miR‐148a, and AFP had the best diagnostic power: AUC 0.931 (95% CI: 0.857–0.973, *P* < 0.0001), with 86.0% sensitivity and 87.5% specificity (Fig. 4E). The established diagnostic model was *y* = −2.512 − 0.485 × miR‐122 + 191.815 × miR‐148a + 0.036 × AFP. And Figure 4F–I showed the ROC curves of miR‐122, miR‐148a, miR‐1246, and AFP for distinguishing early HCC from LC. Among them, miR‐148a was significantly superior to AFP (*P* < 0.05), and still the combination of miR‐122, miR‐148a, and AFP (Fig. 4J) was the best diagnostic model with AUC 0.947 (95% CI: 0.859–0.988, *P* < 0.0001), 87.0% sensitivity and 90.0% specificity (the diagnostic model: *y* = −3.492 − 0.892 × miR‐122 + 245.009 × miR‐148a+ 0.046 × AFP).

**Figure 5:**
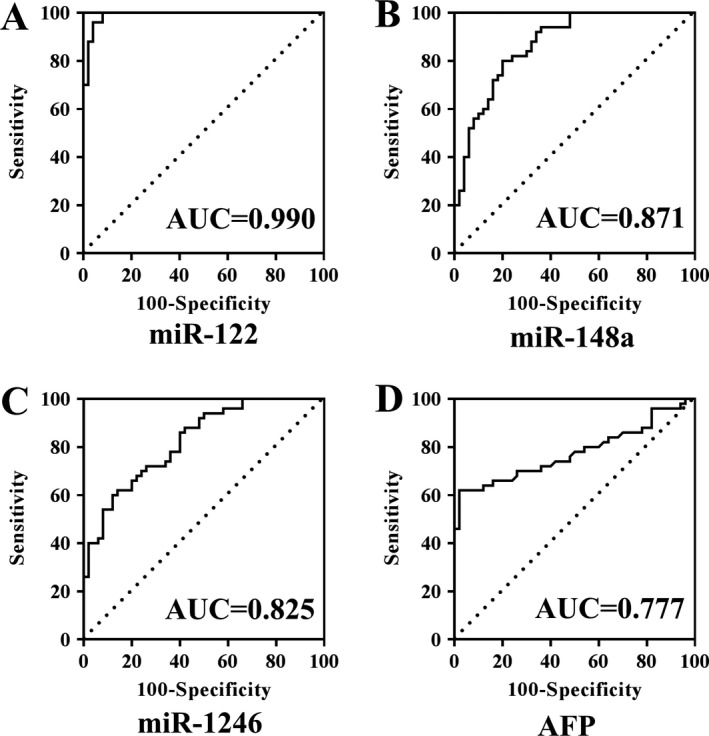
ROC curve analysis of serum exosomal microRNAs to differentiate patients with HCC (*n* = 50) from NC (*n* = 50). miR‐122 (A), miR‐148a (B), miR‐1246 (C), and AFP (D).

To distinguish HCC from NC, serum exosomal miR‐122 had the highest diagnostic value with AUC 0.990 (95% CI: 0.945–1.000, *P* < 0.0001), 100% sensitivity and 92.0% specificity (Fig. 5). At the final validation step, none of the above three serum exosomal microRNAs was differently expressed between HCC and CH groups. Therefore, some useful and accurate diagnostic tool for discriminating HCC from CH requires further detailed study.

### Correlations between exosomal microRNAs and laboratory parameters

To figure out whether elevated exosomal microRNAs were correlated with traditional parameters related to HCC such as AFP, ALT, AST, and GGT, linear regression analysis was carried out. As shown in Figure S3A, AFP concentrations in 50 HCC patients increased, but no apparent elevation was found in serum exosomal miR‐122, miR‐148a, and miR‐1246 levels. Similar results were obtained for miR‐122, miR‐148a, and miR‐1246 compared with ALT (Fig. S3B), AST, and GGT (data not shown). The correlation between the overexpression of miR‐122, miR‐148a, and miR‐1246 and the etiology of the patients (HBV, HCV, and normal) were analyzed, and these three exosomal miRNAs were all significantly higher in HBV‐HCC than in HCV‐HCC and NonBNonC‐HCC (normal) groups (*P* < 0.05), not different between HCV‐HCC and NonBNonC‐HCC groups (data not shown).

## Discussion

Recent studies have proved the potential of exosomal microRNAs in circulation as the biomarker for various diseases including cancer. As we now know, this research is the first to focus on the serum exosomal microRNA profiling between LC and HCC with LC individuals using HiSeq sequencing technology. We found that HCC individuals had significantly higher levels of serum exosomal miR‐122, miR‐148a, and miR‐1246. And the combination of exosomal microRNAs and AFP yielded a better diagnostic power than AFP in discriminating subjects with (early) HCC from LC.

For now, it is still challenging and urgent to find more accurate and noninvasive biomarkers for HCC screening and diagnosis. As for it, scientific experiment designs need to be established, and it is necessary to pay attention to follow: (1) the population at high risks, such as the liver cirrhosis patients; (2) the lack of reliable biomarkers for HCC screening or early diagnosis; (3) the difficulty in determining the nature of liver nodules detected by imaging; (4) the important clinical implications of HCC diagnosis at early stage. More and more studies focus on circulating microRNA profile as diagnostic marker due to its stability and repeatability; however, microRNA signature specific for HCC is not consistent presently. This problem may be attributed to the selection of different experiment designs, analytical methods, internal or external control genes, sample types and size, the control cohort (healthy individuals, chronic hepatitis, liver cirrhosis), etiology factors (HBV or HCV infection or both), risk factors (virus infection, alcohol, aflatoxin, and so on), and normalization of qPCR data 23. The present study consisted of discovery step (microRNA sequencing) and validation step (RT‐qPCR analysis). Liver cirrhotic patients are the main control subjects, besides, NC and CH patients are also included. Serum exosomes were used as tested objects, while in most other studies, serum or plasma was frequently used.

Compared to cell‐free fluids such as serum, the application of exosomes has some superiorities for discovering diagnostic biomarker. In fact, exosomes can selectively pack and carry specific cargo (including microRNAs) representing their cells of origin or specific disorders, and its production increased in patients with cancer compared with the healthy individuals 24, 25. Then, researchers have proved that exosomes can protect their containing microRNA from RNase enriched in circulation, which means that exosomal microRNAs are more stable 26. Last but not least, isolation of exosomal microRNA in circulation would reduce or eliminate interference of insignificant nonexosomal microRNA from circulation in both HCC and LC (or NC) subjects. All these aspects allow exosomal microRNA profiling more feasible and reliable for diagnostic performance.

It has been reported that miR‐122 is the most abundant microRNA in the liver, accounting for 70%. Several studies have shown that miR‐122 as the tumor suppressor was downregulated in HCC cancer cell lines and tumor tissues 27, while recent reports demonstrated serum miR‐122 level was higher in HCC than healthy controls and reduced after surgery 28, 29. In accordance with these findings, our data showed exosomal miR‐122 in HCC serum significantly upregulated compared to LC and NC. The results seemed to be opposite but could be postulated that low abundance of miR‐122 in tumor tissues was ascribed to its increased release into blood, probably via exosomes in part. HBV infection can induce miR‐122 secretion from the liver and increase in plasma 30. All the above mechanisms might cause the reduction in miR‐122 in the liver cell and contribute to HCC formation and progression through the activation of Wnt/beta‐catenin pathway. 27 Circulating miR‐122 expression is associated with hepatic necrosis‐inflammation and cell death in patients with chronic viral hepatitis 31. Therefore, its high level in circulation could be due to the massive release from necrotic hepatocyte. This might be the reason for the high exosomal miR‐122 level in serum of CH group. It should be noted that we detected the microRNAs expression of serum exosomes instead of serum; however, the relationship between their serum and exosome expression levels remains unclear. Some researchers have proposed that part of circulating microRNA free of exosomes or multivesicular bodies is stable in blood through binding proteins such as Ago2 32. And our results need further research to clarify.

Up to now, less research on the relation between exosomal miR‐148a and cancer including HCC has been carried out. miR‐148a was reported to be downregulated in HCC tissues compared with adjacent normal tissues, and its downregulation was correlated with poorer overall survival and recurrence‐free survival 33, 34. Wang et al. 35 reported that serum levels of miR‐148/152 were higher in HCC than the healthy control group. However, another report revealed serum miR‐148a level was remarkably upregulated in patients with HCC recurrence after liver transplantation compared with those without and healthy individuals 36. Consistent with them, our data showed that serum exosomal miR‐148a was significantly higher in individuals with HCC than LC and NC. The contradictory results might be due to the difference in the test sample. Less research has been carried out on the relations between miR‐148a and chronic hepatitis, and the reason of its high exosomal level in serum of CH patients is unclear.

At present, miR‐1246 has been revealed to be overexpressed in various cancers, suggesting its oncogenic role 37, 38. In HCC, miR‐1246 could target tumor suppressor CADM1 to promote cell migration 39. Furthermore, Stella Chai discovered that exosomal miR‐1246 was remarkably upregulated in plasma of HCC patients than healthy individuals and could activate Wnt/*β*‐actin signaling to promote tumor progression through Oct4/miR‐1246 axis in HCC cancer stem cells 40. Similarly, our results showed that serum exosomal miR‐1246 level was significantly higher in subjects with HCC than NC and LC, but not different with CH. Perhaps exosomal miR‐1246 are involved in HCC development through promoting cancer stemness. On the contrary, miR‐1246 has been reported to target and downregulate NFIB to inhibit cell proliferation of HCC 41. Given the above, miR‐1246 seems to have two sides: oncogenic and tumor‐suppressive microRNA. Genetic heritage and microenvironment were perhaps also two of the most determinants for tumor development as well as the differences in study design and techniques.

In conclusion, miR‐122, miR‐148a, and miR‐1246 were significantly elevated in serum exosomes from HCC patients compared to LC and NC individuals. Further promotion and use of this method would improve diagnostic performance to enlarge patient cohorts and will be helpful for predicting disease progression and preventive treatment of HCC. The determination method was sensitive and could be applied to a cost‐effective, minimally invasive, and complementary test for detecting HCC in a clinical setting. Although our findings need further research, exosomal microRNAs or their combination with traditional biomarker may serve as promising biomarkers for HCC screening and diagnosis.

## Conflict of Interest

The authors declare no conflict of interests on competing financial and contribution.

## Supporting information


**Figure S1.** Serum exosomal microRNA profiling through Illumina HiSeq 2000 technology. (A) The Scatter plot and (B) volcano plot for the upregulated and downregulated exosomal microRNAs in serum between HCC and LC patients. The vertical lines and horizontal line in Figure 2B represent 1.5‐fold change in up (red points) and down (green points) expression and a *P*‐value of 0.05. The black points in the plot represent microRNA without statistical differences.
**Figure S2.** The amplification curves of the control gene (A) and two target genes with the most abundant and lowest expression (B) in our study.
**Figure S3.** Correlations between serum exosomal microRNAs and AFP (A) and ALT (B) in patients with HCC.

**Table S1.** Basic information of 10 cases for microRNA sequencing. Ages are given as means ± SD.
**Table S2.** Clinical characteristics of 55 cases. Ages are given as means ± SD.
