# Supplementary material for: Serum exosomal microRNAs combined with alpha‐fetoprotein as diagnostic markers of hepatocellular carcinoma
Source: Cancer Med. 2018 Mar 23;7(5):1670–9. doi: 10.1002/cam4.1390 (PMC5943469; doi:10.1002/cam4.1390)
Supplement: Supplementary file 1 — Figure S1. Serum exosomal microRNA profiling through Illumina HiSeq 2000 technology. (A) The Scatter plot and (B) volcano plot for the upregulated and downregulated exosomal microRNAs in serum between HCC and LC patients. The vertical lines and horizontal line in Figure 2B represent 1.5‐fold change in up (red points) and down (green points) expression and a P‐value of 0.05. The black points in the plot represent microRNA without statistical differences. Figure S2. The amplification curves of the control gene (A) and two target genes with the most abundant and lowest expression (B) in our study. Figure S3. Correlations between serum exosomal microRNAs and AFP (A) and ALT (B) in patients with HCC. [file CAM4-7-1670-s001.pdf]

Fig. S1

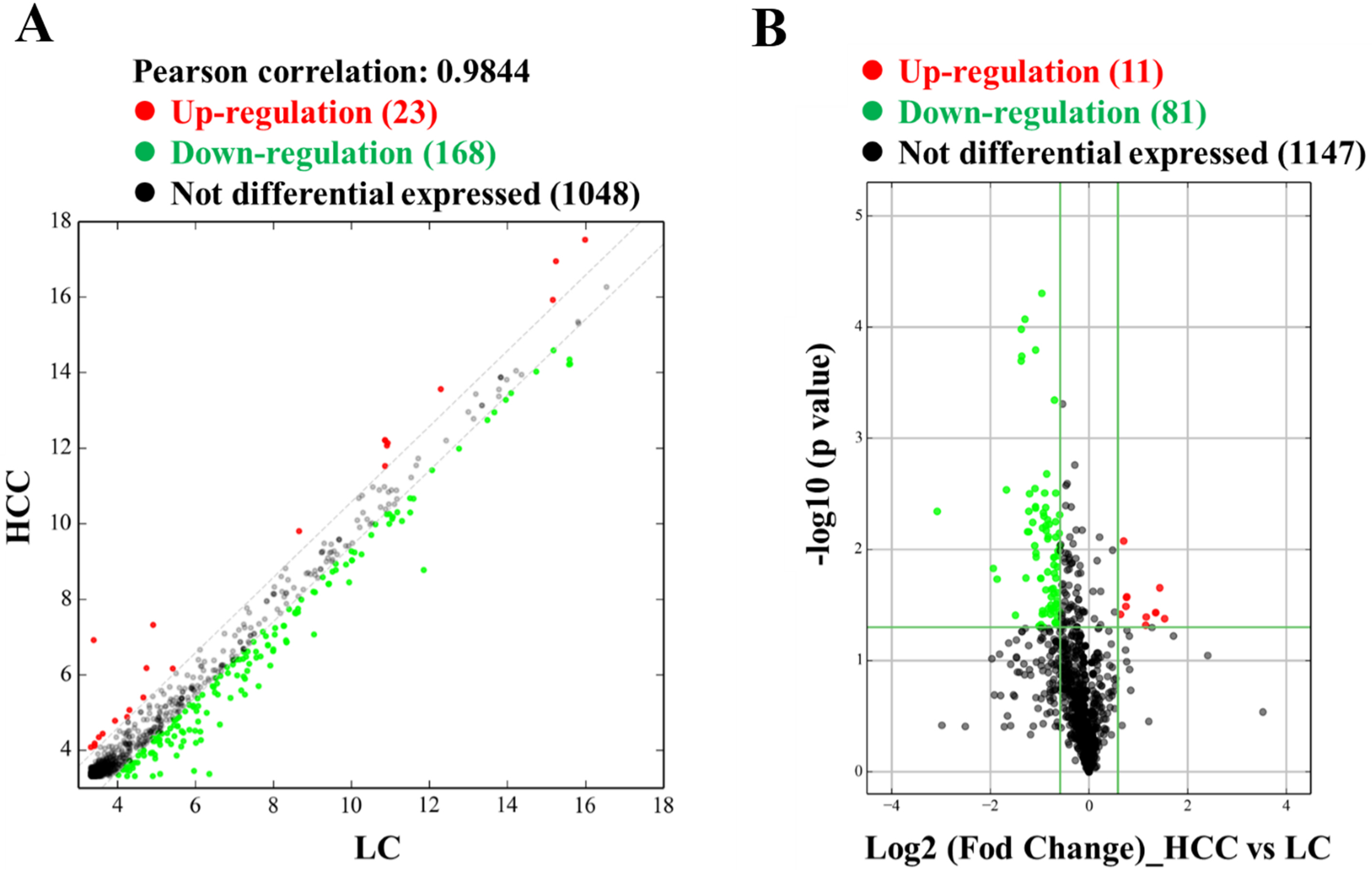
A
Pearson correlation: 0.9844
Up-regulation (23)
Down-regulation (168)
Not differential expressed (1048)
HCC
LC
4
6
8
10
12
14
16
18
B
Up-regulation (11)
Down-regulation (81)
Not differential expressed (1147)
-log10 (p value)
Log2 (Fod Change)_HCC vs LC
0
1
2
3
4
5
-4
-2
0
2
4

Fig. S2

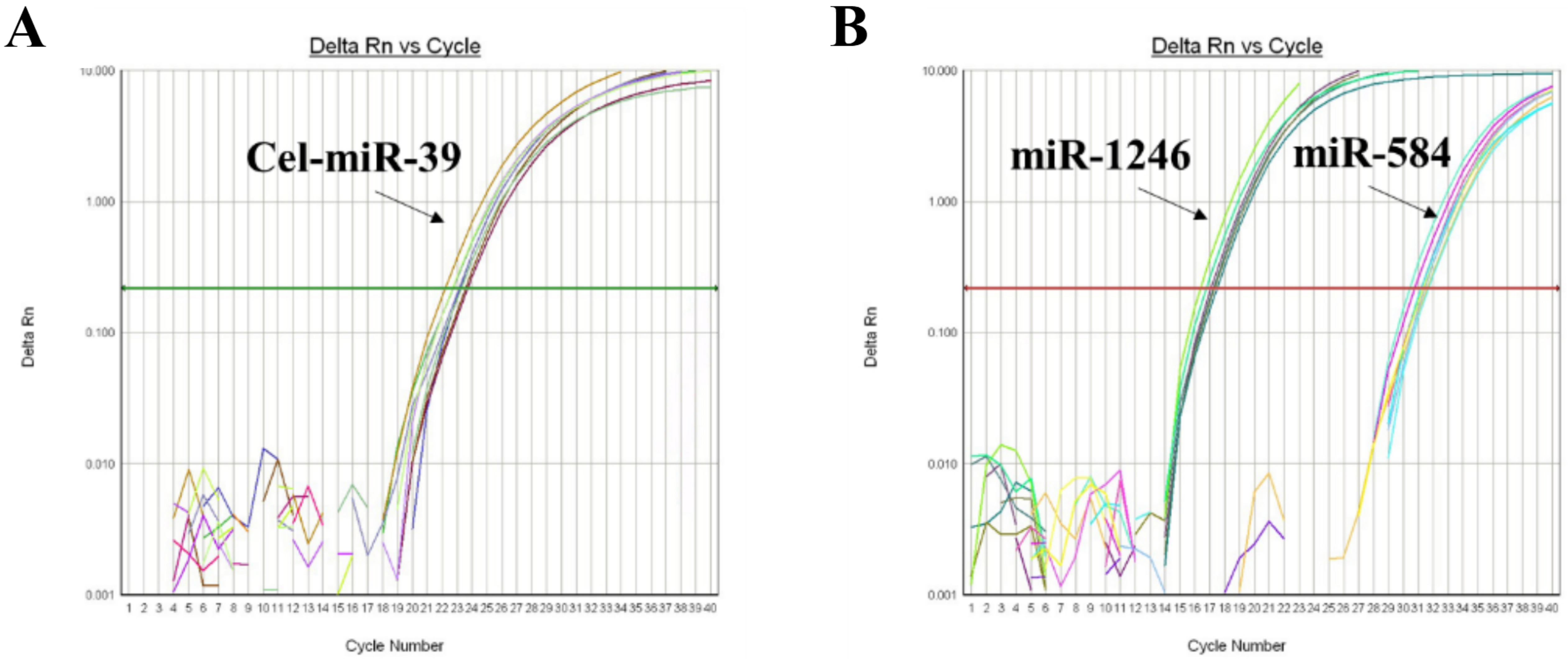
A
Delta Rn vs Cycle
Cel-miR-39
Delta Rn
10.000
1.000
0.100
0.010
0.001
1 2 3 4 5 6 7 8 9 10 11 12 13 14 15 16 17 18 19 20 21 22 23 24 25 26 27 28 29 30 31 32 33 34 35 36 37 38 39 40
Cycle Number
B
Delta Rn vs Cycle
miR-1246
miR-584
Delta Rn
10.000
1.000
0.100
0.010
0.001
1 2 3 4 5 6 7 8 9 10 11 12 13 14 15 16 17 18 19 20 21 22 23 24 25 26 27 28 29 30 31 32 33 34 35 36 37 38 39 40
Cycle Number

Fig. S3

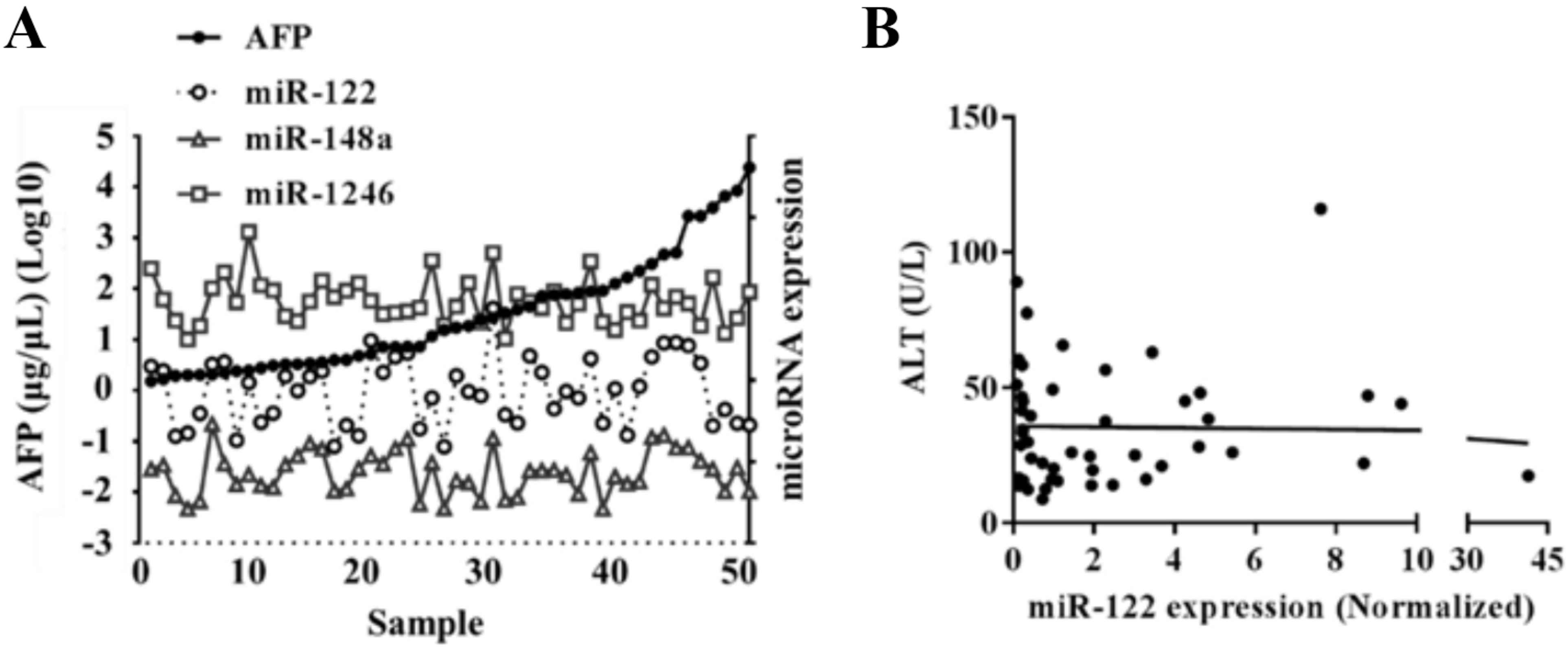
A
AFP
miR-122
miR-148a
miR-1246
AFP (μg/μL) (Log10)
microRNA expression
Sample
B
ALT (U/L)
miR-122 expression (Normalized)
