# Supplementary material for: Serum exosomal microRNAs combined with alpha‐fetoprotein as diagnostic markers of hepatocellular carcinoma
Source: Cancer Med. 2018 Mar 23;7(5):1670–9. doi: 10.1002/cam4.1390 (PMC5943469; doi:10.1002/cam4.1390)
Supplement: Supplementary file 2 — Table S1. Basic information of 10 cases for microRNA sequencing. Ages are given as means ± SD. Table S2. Clinical characteristics of 55 cases. Ages are given as means ± SD. [file CAM4-7-1670-s002.docx]

Table S1 Basic information of 10 cases for microRNA sequencing. Ages are given as means ± S.D..

| **Variables** | **HCC (n=5)** | **LC (n=5)** | ***p*-value** |
| --- | --- | --- | --- |
| Age, years (mean ± SD) | 56.40±1.95 | 56.00±1.58 | NS |
| Sex, male/female (n) | 5/0 | 5/0 | NS |
| Etiology,HBV/HCV/Normal (n) | 5/0/0 | 5/0/0 | NS |
| AFP, >20/<20 ng/ml (n) | 1/4 | 0/5 | NS |

NS: no difference between groups

Table S2 Clinical characteristics of 55 cases. Ages are given as means ± S.D..

| **Variables** | **HCC**  **(n=18)** | **LC**  **(n=13)** | **CH**  **(n=10)** | **NC**  **(n=14)** | ***p*-value** |
| --- | --- | --- | --- | --- | --- |
| Age, years | 55.50±13.67 | 51.62±15.26 | 50.10±6.72 | 52.79±4.28 | NS |
| Sex, male/female (n) | 16/2 | 9/4 | 8/2 | 8/6 | NS |
| AFP, >20/<20 ng/ml (n) | 14/4 | 2/11 | 0/10 | 0/14 | <0.001*** |

AFP, alpha fetoprotein; CH, chronic hepatitis; HCC, hepatocellular carcinoma; LC, liver cirrhosis; NC, normal; NS, no difference among groups. ***P < 0.001.
